# Caffeine consumption and telomere length in men and women of the National Health and Nutrition Examination Survey (NHANES)

**DOI:** 10.1186/s12986-017-0162-x

**Published:** 2017-01-31

**Authors:** Larry A. Tucker

**Affiliations:** College of Life Sciences, Brigham Young University, 237 SFH, Provo, UT 84602 USA

**Keywords:** Cell aging, Coffee, Stimulant, Leukocyte, DNA

## Abstract

**Background:**

The investigation evaluated the relationship between caffeine intake and coffee consumption and leukocyte telomere length, a biomarker of the senescence of cells.

**Methods:**

A total of 5826 adults from the National Health and Nutrition Examination Survey (NHANES) were studied cross-sectionally. Using the quantitative polymerase chain reaction method, telomere length was compared to standard reference DNA. Caffeine intake from foods and beverages and coffee consumption were measured using a validated, multi-pass, computer-assisted, 24-h recall system administered by NHANES interviewers. The following covariates were controlled: age, gender, race, marital status, education, housing, smoking, BMI, physical activity, alcohol use, and coffee intake (or caffeine consumption).

**Results:**

Caffeine consumption was inversely related to telomere length (*F* = 15.1, *P* = 0.0005). For each 100 mg of caffeine consumed, telomeres were 35.4 base pairs shorter, after adjusting for the covariates. For each 100 mg of caffeine consumed among coffee drinkers only, telomeres were 36.7 base pairs shorter (*F* = 9.0, *P* = 0.0054), and among non-coffee drinkers only, 40.0 base pairs shorter (*F* = 8.5, *P* = 0.0067). Conversely, coffee intake was positively related to telomere length (*F* = 12.6, *P* = 0.0013), independent of the covariates.

**Conclusions:**

Results suggest that caffeine consumption accounts for shorter telomeres in U.S. adults, independent of numerous covariates, whereas coffee intake predicts longer telomeres.

## Background

Caffeine is one of the most commonly consumed drugs in the world [1]. Many popular beverages, such as coffee, tea, soda, and energy drinks contain caffeine, as well as other products, such as chocolate and medications. Because of caffeine’s significant physical, metabolic, and psychoactive properties, there is considerable public health interest in its effects on humans.

Caffeine intake has been linked to a number of beneficial and detrimental health consequences [1]. However, much of the research on the effects of caffeine has focused on the consumption of coffee, and other beverages that contain caffeine, but not specifically caffeine.

The relationship between coffee consumption, disease, and mortality has been studied many times. One of the most recent and largest investigations was conducted using the Health Professionals Follow-up data and the Nurses’ Health Study data combined [2]. In this investigation, coffee consumption was inversely related to all-cause mortality, mainly due to a moderately reduced risk of cardiovascular disease (CVD) mortality. However, the significant association was independent of caffeine intake. In fact, decaffeinated coffee intake was also associated with reduced risk of all-cause and CVD mortality, indicating that components of coffee other than caffeine are likely driving the coffee and mortality connection. Other investigations indicate that the beneficial effects of coffee are likely based on its anti-inflammatory properties [3, 4], and the authors of this large epidemiologic study [2] suggest that the apparently healthy components of coffee offset some of the potential harmful effects of caffeine [5–7].

A similar finding was revealed by Loftfield et al in a 2015 publication [8]. All-cause mortality tended to decrease as coffee consumption increased, but decaffeinated coffee showed comparable results, suggesting that factors other than caffeine are likely responsible for the favorable link between coffee drinking and mortality.

Although the associations between coffee intake, disease, and mortality have been investigated multiple times, research evaluating the relationship between caffeine intake and telomere length relationship is extremely rare. In 2016, Liu et al showed that higher coffee consumption in female nurses was related to longer telomeres. However, after adjusting for potential confounders, caffeine consumption was not predictive of telomere length. Clearly, the coffee and telomere versus caffeine and telomere links are not the same.

Of particular interest are the potential clastogenic effects of caffeine. Because caffeine tends to inhibit cell cycle-dependent DNA repair, inducing potential disruption of chromosomes [9–11], accelerated biologic aging is a potential consequence. Telomere length is a molecular index of biologic aging [12, 13]. Research focusing on a nationally representative sample, such as NHANES, could provide valuable insights regarding the relationship between caffeine consumption and telomere length in adults.

The primary purpose of the present study was to determine the magnitude and intricacies of the relationship between caffeine intake and leukocyte telomere length using data from NHANES, a large, ethnically and socioeconomically varied, heterogeneous sample of U.S. men and women. Secondary objectives were to investigate the association between coffee intake and telomere length, and to evaluate the potential effects of several covariates, including age, gender, race, marital status, education, housing, smoking, body mass index (BMI), physical activity, alcohol use, and coffee intake on the relationship between caffeine consumption and leukocyte telomere length.

### Aging and telomere length

Repeated at each chromosome end, telomeres are nucleoprotein structures that protect the ends of euraryotic chromosomes. The caps are comprised of a simple sequence of telomeric DNA, TTAGGG. With mitosis, telomeres naturally shorten. Part of the telomeric DNA does not replicate each time a cell divides. This is commonly referred to as the “end replication problem,” and short telomeres generally lead to negative health consequences [14].

Before mitotic cells become old and lose their ability to divide, they undergo a limited number of cell divisions. Hence, telomere length is a biomarker of both the replicative past and the replicative potential of somatic cells. Evidence is mounting in support of the notion that telomeres play a significant part in the senescence and destiny of cells [14]. Research by Weischer et al. [15], including nearly 20,000 participants, showed that individuals with telomeres in the shortest category had 25% greater risk of early death compared to those in the longest telomere category, after adjusting for potential confounders. Likewise, Njajou et al. [16] showed that telomere length was predictive of years of healthy life, and Carty et al. [17] found that shorter telomere length was associated with higher mortality in women.

## Methods

### Sample

For more than 50 years, the National Health and Nutrition Examination Survey has been conducted by the Centers for Disease Control and Prevention to provide estimates of the health and nutritional status of non-institutionalized civilians living in the United States. For data collection, NHANES employs a complex, multistage, probability sampling design [18]. In stage 1, primary sampling units (PSUs) are selected. These are mostly single counties. For stage 2, PSUs are divided into segments, generally city blocks or their equivalent. In stage 3, households within each block are chosen randomly. Finally, in stage 4, participants are selected from a list of all individuals residing in selected households. Individuals are drawn at random within age-sex-race screening subdomains [18].

NHANES data containing telomere length measures are available for a 4-year period only, 1999–2002. These data became available to the public in November, 2014. NHANES 1999–2002 oversampled individuals 12–19 years, persons age 60 and older, African Americans, Mexican Americans, and low-income individuals to provide more accurate estimates of these groups. All of the data are cross-sectional [18]. The NHANES data sets used by the present study are available to the public online at no cost [19].

DNA samples were requested from all respondents of NHANES 1999–2000 and NHANES 2001–2002, ages 20 years and older. Of the 10,291 participants who were eligible to give DNA samples, 3567 adults from NHANES 1999–2000 and 4260 from NHANES 2001–2002 consented to have their DNA used for future research and provided a useable DNA sample (*n* = 7827, 76%). Because survival bias is a potential issue among the extremely old, and because individuals 85 years and older were all given the age of 85 by NHANES, participants 85 years old and older were excluded from the data set. Only participants with complete data, including values for telomere length, caffeine and coffee consumption, and the potential mediating variables, were included in the analyses, a total of 5826 participants, 2741 men and 3085 women. The National Center for Health Statistics Ethics Review Board at the Centers for Disease Control and Prevention approved collection of the NHANES data and posting of the NHANES public-use files, which were required for this investigation. All NHANES participants provided written informed consent.

### Measures

Data were collected on 13 variables in the present investigation: leukocyte telomere length, caffeine intake, age, gender, race, education, marital status, housing, body mass index (BMI), smoking, physical activity, alcohol use, and coffee intake.

#### Telomere length

The procedure used to measure telomere length has been described in detail by NHANES [20] and elsewhere [21, 22]. Blood samples were collected as part of the NHANES survey and used for DNA analysis. According to NHANES [20], DNA was extracted from the samples and stored at -80°C at the Division of Health Sciences Laboratory, CDC. Specimens were then shipped to the laboratory of Dr. E. Blackburn (University of California, San Francisco) for analysis. Using the quantitative polymerase chain reaction method, leucocyte telomere length was measured and compared to standard reference DNA (T/S ratio). Five 96-well quality control plates, representing 5% of the complete set, were provided. The investigators were blinded regarding the duplicate samples. Each specimen was assayed 3 times on 3 different days. Each specimen was assayed using duplicate wells resulting in 6 values. The six measurements were used to determine the mean and standard deviation of the T/S ratio. Plates containing the samples were assayed in groups of 3, with no 2 plates grouped together more than once. Control DNA measures were employed to normalize between-run variability. If more than 5% of the duplicate samples on the quality control plates were discordant with their pair in the complete set, the variant failed the quality control. For each sample, potential outliers were flagged and excluded from calculations (<2% of samples). The inter-assay coefficient of variation was 6.5% [20]. Mean T/S ratio values were converted to base pairs using the formula: 3274 + 2413 × (T/S). It is important to note that base pair estimates are only comparable for T/S ratio data produced using the same reference standard and the same lab procedures [20].

#### Caffeine and coffee consumption

According to NHANES [23], dietary intake data were collected via a 24-h dietary recall using a computer-assisted dietary interview system, which was administered by an NHANES interviewer. Many studies have used the NHANES 24-h dietary recall system [24–26]. Interviewers were bilingual, trained, and each had a college degree in Food and Nutrition or Home Economics, with at least 10 credits in food and nutrition. Interviews were conducted in a private setting in an NHANES Mobile Examination Center [23].

The dietary assessment was used to collect detailed information about all foods and beverages consumed, including a complete description of each food and the amount consumed. Nutrients and non-nutrient food components, including caffeine intake and coffee consumption, were calculated from foods and beverages that were consumed during the 24-h period prior to the interview (midnight to midnight). Coffee consumption included all forms of coffee. The interview used a multi-pass format. Food probes that were used in previous NHANES and USDA surveys were part of the built-in features of the system. The computer-assisted system provided a standardized interview format. Interviewers followed scripts provided in the system to explain the dietary interview component to the participant [27].

#### Covariates

NHANES used five categories to differentiate among races and ethnicities: Non-hispanic White, Non-hispanic Black, Mexican American, Other race or Multi-racial (Other), and Other Hispanic. Education level was defined using three categories: Less than high school, high school diploma (including GED), and more than high school. Marital status was defined by seven categories: married, widowed, divorced, separated, never married, living with partner, or other. Housing arrangements, a measure of social economic status, was defined using three categories: home owned or being bought, home being rented, and other.

Several lifestyle factors were measured in the present study to serve as potential mediating variables. Participation in leisure time physical activity was quantified using met-minutes of activity per week during the past 30 days. Specifically, participants were asked to report from a list, which of 62 physical activities they participated in regularly, whether the activity was moderate or vigorous, the number of times in the past 30 days they engaged in the activity, and the average duration of the activity. Durations of less than 10-min were not counted. A MET score was produced for each activity and total MET-minutes per week were calculated by NHANES for each participant using the compendium of physical activities [28]. Although a majority of the sample was sedentary, non-sedentary participants were divided into sex-specific tertiles, resulting in four categories of physical activity: sedentary, low, moderate, and high.

Smoking was indexed using a measure of pack-years, representing cumulative exposure to tobacco smoke. Pack years were calculated as the mean number of cigarettes smoked per day times the number of years smoked, divided by 20.

Body mass index (BMI) represents participants’ weight in kilograms divided by the square of their height in meters. BMI (kg/m^2^) allows the comparison of body weights independent of height. BMI categories were used to differentiate among participants who were underweight (<18.5), normal weight (≥18.5 and < 25.0), overweight (≥25.0 and < 30.0), obese (≥30.0) or missing.

Three categories were used to define alcohol use: abstainers, moderate drinkers, and heavy drinkers. Abstainers were those reporting no alcohol use in the past 12 months. Moderate drinkers were defined as women who reported drinking more than 0 but less than 2 drinks per day over the past 12 months, or men who reported drinking more than 0 and less than 3 alcoholic beverages per day during the past 12 months. Heavy drinkers were women who reported drinking 2 or more alcoholic beverages per day over the past 12 months or men who reported drinking 3 or more alcoholic drinks per day over the past 12 months.

### Statistical analysis

NHANES assigns an individual-level sample weight to each participant [29]. It is a measure of the number of people in the population represented by that sample-person in NHANES, reflecting the unequal probability of selection, nonresponse adjustment, and adjustment to independent population controls. When unequal selection probability is applied, the sample weights are used to produce an unbiased national estimate. For the present study, sample weights were based on 4 years of diet data, which included the caffeine and coffee consumption variables.

In the present study, frequencies for categorical variables, and means (±SE) for continuous variables, were reported to describe the data. Each descriptive value included adjustments based on the complex sampling design of NHANES by incorporating strata, primary sampling unit (PSU) indicators, and sample weights [29]. The SAS SurveyMeans procedure was employed to generate weighted means that represent values for the U.S. population, and SAS SurveyFreq was used to calculate weighted frequencies, which are also generalizable to the U.S. adult population.

For the current study, total caffeine consumption (mg per day) was the primary exposure variable. Coffee consumption (g per day) was also evaluated as a secondary exposure variable. Coffee intake was measured in grams by NHANES. To convert to cups, grams of coffee can be divided by 225.

The extent of the linear associations between caffeine intake and telomere length and coffee consumption and telomere length were measured using regression analysis and the SAS SurveyReg procedure. Regression estimates for each model were based on the complex, multistage, probability sampling process of NHANES. To test the extent to which the relationships between the exposure variables and telomere length were mediated by differences in age, race, marital status, education, housing, smoking, alcohol use, body mass index, physical activity, and coffee consumption (or caffeine intake), these factors were controlled statistically using partial correlation and the SAS SurveyReg procedure. Throughout the paper, statements that adjustments were made for “the covariates” indicates that all these covariates were controlled simultaneously using partial correlation.

Separate models were used to determine the magnitude of the linear relationship between caffeine intake and telomere length among all participants, coffee drinkers only, and those reporting no coffee intake. When treated as a categorical variable, coffee intake was divided into four groups. Quartiles could not be used to divide coffee intake because 48% of the subjects reported no coffee consumption. Beyond those reporting no coffee intake, coffee drinkers were divided into tertiles, resulting in four categories: 1) no coffee intake, 2) 1–355 g, 3) 356–639 g, 4) 640 or more grams.

To study the relationships between telomere length and the covariates, including coffee intake, mean differences in telomere length were compared across each level of the covariates. Additionally, to afford an additional perspective of the caffeine intake and telomere length relationship, mean differences in telomere base pairs were tested across four categories of caffeine consumption based on 150 mg increments: 0 mg, 1–149 mg, 150–299 mg, 300–449 mg, and 450 mg or more per day. Mean telomere differences across the caffeine categories were studied within three separate samples: all participants, coffee drinkers only, and those reporting no coffee intake.

All *P*-values were two-sided and statistical significance was accepted when alpha was < 0.05. Statistical analyses were performed using SAS Version 9.4 (SAS Institute, Inc., Cary, NC).

## Results

Using sample weights to generate results representative of the U.S. non-institutionalized population, mean (±SE) age of the sample was 46.9 ± 0.5 years. Mean caffeine intake from foods and beverages was 195 ± 9 mg per day, and mean coffee intake for the sample was 342 ± 21 g (1.5 ± 0.1 cups) per day. Additionally, average telomere length was 5828 ± 40 base pairs. Table 1 shows weighted percentiles (±SE) for telomere length (base pairs) and caffeine consumption (mg per day). The table values represent those of the U.S. adult population.

**Table 1:** Percentiles for telomere length (base pairs) and caffeine intake among U.S. women and men

	Percentile (±SE)				
Variable	5th	25^th^	50^th^	75^th^	95^th^
Telomere Length (base pairs)					
Women (*n* = 3085)	4937 ± 42	5386 ± 42	5743 ± 40	6178 ± 54	7007 ± 111
Men (*n* = 2741)	4948 ± 30	5353 ± 31	5735 ± 39	6193 ± 50	7022 ± 82
Combined (*n* = 5826)	4943 ± 32	5373 ± 33	5738 ± 37	6188 ± 49	7014 ± 85
Caffeine Intake (mg per day)					
Women (*n* = 3085)	0 ± 0.4	13 ± 7	109 ± 9	231 ± 11	555 ± 24
Men (*n* = 2741)	0 ± 0.6	47 ± 9	145 ± 11	302 ± 14	685 ± 30
Combined (*n* = 5826)	0 ± 0.4	32 ± 9	124 ± 8	262 ± 9	616 ± 30

*SE* standard error. Table values include person-level weighted adjustments based on the sampling design of NHANES so that values reflect those of the U.S. population

In the present study, as expected, age and telomere length were strongly and inversely related. Specifically, the model-based estimate of the age-related rate of telomere shortening was 15.3 base pairs per year (*F* = 441.4, *P* < 0.0001). Beyond the linear term, age-squared was not associated with telomere length (*F* = 0.2, *P* = 0.6478).

Table 2 displays the weighted descriptive characteristics of the sample, with focus on the covariates.

**Table 2:** Descriptive characteristics of the sample and mean leukocyte telomere length (base pairs) across each level of the categorical covariates of the study

Variable	N	Weighted %	Adjusted* Telomere mean	SE	F	P
Gender					2.5	0.1267
Men	2741	47.4	5893	51		
Women	3085	52.6	5927	49		
Race					4.0	0.0103
Non-Hispanic White	2902	73.1	5891^a,c^	62		
Non-Hispanic Black	1056	10.4	6012^b^	72		
Mexican American	1428	7.3	5790^a^	51		
Other Race	144	3.1	5891^a,b,c^	68		
Other Hispanic	296	6.1	5972^b,c^	64		
Education					3.3	0.0529
< High School	2068	22.8	5862	50		
High School Diploma	1356	26.0	5921	57		
> High School	2402	51.2	5950	52		
Marital Status					2.0	0.0967
Married	3323	57.0	5901	46		
Widowed	492	6.2	5919	63		
Divorced	484	9.3	5877	58		
Separated	201	3.1	5785	61		
Never Married	821	15.4	5935	65		
Living with Partner	313	5.8	5852	59		
Other	192	3.3	6108	100		
Housing Status					6.3	0.0054
Own or Buying	3824	69.8	5848^a^	39		
Renting House	1841	28.0	5849^a^	49		
Other	161	2.2	6036^b^	77		
Physical Activity					6.1	0.0024
Sedentary	3375	49.5	5860^a^	43		
Low Activity	812	15.4	5885^a^	51		
Moderate Activity	821	17.3	5890^a^	63		
High Activity	818	17.8	6010^b^	63		
Body Mass Index					4.1	0.0094
Missing	149	2.2	5983^a^	77		
Underweight	83	1.7	5946^a,b^	79		
Normal Weight	1611	30.3	5924^a^	46		
Overweight	2066	33.6	5861^a,b^	54		
Obese	1917	32.1	5842^b^	46		
Alcohol Use					1.2	0.3129
Abstainer	2312	36.2	5894	44		
Moderate Drinker	1776	31.9	5899	56		
Heavy Drinker	1738	31.9	5940	57		
Coffee Intake					4.5	0.0104
None	2802	48.1	5912^a,b^	46		
Low	1175	17.2	5877^b^	59		
Moderate	1024	17.4	5901^b^	48		
High	825	17.3	5997^a^	71		

* Telomere means were adjusted for differences in all of the covariates

^a,b,c^ Telomere means across different levels of the same variable with the same superscript letter are not significantly different. Age and smoking (pack-years) were treated as continuous variables and therefore are not part of Table 2. All Ns are unweighted and all proportions are survey-weighted. Focus should be on the survey-weighted proportions because they represent the U.S. adult population

Table 3 shows the magnitude of the linear associations between caffeine intake and telomere length and between coffee intake and telomere length, after adjusting for the covariates. In Table 3, caffeine intake, coffee consumption, and telomere length were each treated as continuous variables. Caffeine intake was inversely and linearly related to telomere length. Among all participants (*n* = 5826), for each 100 mg of caffeine consumed per day, telomere length was 35.4 base pairs shorter (*F* = 15.1, *P* = 0.0005), after adjusting for the covariates, including age, sex, race, education, marital status, housing, BMI, smoking, physical activity, coffee intake, and alcohol use. Similarly, in a separate model delimited to only coffee drinkers (*n* = 3024), telomere length was 36.7 base pairs shorter for each 100 mg of caffeine consumed per day, after controlling for the covariates (*F* = 9.0, *P* = 0.0054). With the covariates controlled and the sample confined to adults reporting no coffee intake (*n* = 2802), telomere length was 40.0 base pairs shorter for each 100 mg of caffeine consumed (*F* = 8.5, *P* = 0.0067).

**Table 3:** Telomere length as it relates to caffeine intake and coffee consumption in U.S. adults, separated by coffee drinking status, after adjusting for the covariates

	Telomere length (base pairs)			
Exposure variable	Regression			
Sample	Coefficient	SE	F	P
Caffeine intake per 100 mg				
All Participants (*n*=5826)	-35.4	9.1	15.1	0.0005
Coffee Drinkers Only (*n*=3024)	-36.7	12.2	9.0	0.0054
No Coffee Intake Reported (*n*=2802)	-40.0	13.7	8.5	0.0067
Coffee Intake per 100 g (3.55 oz)				
All Participants (*n*=5826)	15.0	4.2	12.6	0.0013
Coffee Drinkers Only (*n*=3024)	17.9	5.9	9.1	0.0053
No Coffee Intake Reported (*n*=2802)	–	–	–	–

Each regression model tested the linear association between the exposure variable, either caffeine or coffee intake, and telomere length, separated by coffee drinking status, after adjusting for the covariates. For the caffeine and telomere length associations, in the samples that included coffee drinkers, age, race, education, marital status, housing, BMI, physical activity (MET-minutes), smoking (pack-years), alcohol use, and coffee intake were controlled statistically. For the coffee and telomere length models, the same covariates were controlled, except adjustments were made for caffeine intake rather than coffee consumption. Interpretation of the first row of regression results should be as follows: After adjusting for the covariates, for each 100 mg of caffeine consumed per day by U.S. adults, telomere length was 35.4 base pairs shorter, on average

Conversely, as shown in Table 3, coffee consumption was positively related to the length of telomeres. Specifically, for each 100 g (3.55 oz) of coffee consumed, telomeres were 15.0 base pairs longer, on average, after adjusting for the covariates (*F* = 12.6, *P* = 0.0013). With consumption of coffee indexed using cups instead grams, telomeres were 33.8 base pairs longer for each cup of coffee consumed. With the sample delimited to only coffee drinkers (*n* = 3024), telomeres were 17.9 base pairs longer for each 100 g or 40.3 base pairs longer per cup of coffee consumed per day, with the covariates controlled (*F* = 9.1, *P* = 0.0053).

Table 4 shows the relationship between caffeine intake and telomere length using a different analysis strategy. Specifically, caffeine intake was treated as a categorical variable, and mean differences in telomere length were compared across five incremental caffeine groups. Mean telomere length differed significantly across the caffeine categories in U.S. adults. Differences in telomere length were significant and dose-response when all participants were studied together and within each of the subsamples, coffee drinkers only and those reporting no coffee intake.

**Table 4:** Differences in mean telomere length (base pairs) by level of daily caffeine intake for all participants, coffee drinkers only, and adults reporting no coffee intake, after adjusting for the covariates

		Caffeine intake					
	Group 0	Group 1	Group 2	Group 3	Group 4		
	0 mg	1–149 mg	150–299 mg	300–449 mg	450+ mg		
Telomere Length:	Mean + SE	Mean ± SE	Mean ± SE	Mean ± SE	Mean ± SE	F	P
All participants (*n* = 5826)	5980^a^ + 59	5941^a^ ± 49	5867^b^ ± 57	5850^b^ ± 72	5701^c^ ± 67	6.1	0.0011
(sample size and %)	(862, 12%)	(2796, 44%)	(1248, 23%)	(493, 10%)	(427, 11%)		
Coffee drinkers (*n* = 3024)	—	5862^a^ ± 78	5803^a,b^ ± 70	5764^b^ ± 99	5606^c^ ± 89	5.0	0.0066
(sample size and %)		(1273, 34%)	(937, 32%)	(418, 16%)	(396, 19%)		
No coffee intake (*n* = 2802)	6079^a^ + 67	6046^a^ + 63	5957^b^ + 74	—	—	3.6	0.0398
(sample size and %)	(661, 24%)	(1524, 56%)	(417, 20%)				

^a,b,c^ Telomere means on the same row with the same superscript letter are not significantly different. Means have been adjusted for differences in the covariates, including age, gender, race, education, marital status, housing, BMI, physical activity, coffee intake, alcohol use, and smoking. Under each mean, in parentheses, the unweighted sample size (n) and the survey-weighted proportion of each subgroup are included. Focus should be on the survey-weighted proportions because they represent the U.S. adult population. In adults with no coffee intake, the sample sizes for caffeine Groups 3 and 4 were insufficient for analysis (*n* = 75 and *n* = 31, respectively). Therefore, they were merged with Group 2. Including all participants, the mean difference in telomere length between Groups 1 and 2 was borderline significant (*P* > 0.05 and *P* < 0.10). Among coffee drinkers only, the difference between Groups 3 and 4 was *P* = 0.054, and in adults reporting no coffee intake, the difference between Groups 1 and 2 was *P* = 0.057

With caffeine intake treated as a continuous variable, given the model-based estimate of the age-related rate of telomere shortening was 15.3 base pairs per year in U.S. adults, adults of the same age, gender, race, education, marital status, housing, BMI, physical activity level, smoking habit, coffee intake, and alcohol use had estimated biological aging differences of about 2.3 years per 100 mg caffeine consumed (35.4÷15.3 = 2.3). As shown in Table 3, among coffee drinkers only, the difference was 2.4 years of accelerated aging per 100 mg of caffeine (36.7÷15.3 = 2.4), and among those reporting no coffee intake, cell senescence was 2.6 years higher for each 100 mg of caffeine consumed (40.0÷15.3 = 2.6).

With coffee intake treated as a continuous variable, for each cup of coffee consumed per day (225 g per cup), cell aging was about 2.2 years less among U.S. adults, after adjusting for the covariates. Among coffee drinkers only, cell senescence was 2.6 years less for each cup of coffee consumed, with the covariates controlled.

With participants separated by gender, the relationship between caffeine intake and telomere length remained significant and inverse. Specifically, after controlling for differences in the covariates, for each 100 mg of caffeine consumed, telomeres were 53.3 base pairs shorter for women (*F* = 8.5, *P* = 0.0068) and 25.4 base pairs shorter for men (*F* = 9.0, *P* = 0.0056). On the other hand, after adjusting for differences in the covariates, for each 100 g (3.55 oz) of coffee consumed, telomeres were 18.0 base pairs longer for women (*F* = 5.7, *P* = 0.0239) and 12.4 base pairs longer for men (*F* = 6.6, *P* = 0.0154).

### Telomere length and the covariates

Most of the potential mediating variables were significantly associated with telomere length, after adjusting for differences in the covariates (Table 2). However, telomere length did not reach significance when comparing women and men (*F* = 2.5, *P* = 0.1267). Race was a significant predictor of telomere length (*F* = 4.0, *P* = 0.0103), after adjusting for the covariates (Table 2). Blacks had longer telomeres than Whites and Mexican Americans. Additionally, Other Hispanics had longer telomeres than Mexican Americans. Telomere differences across education levels were borderline significant (*F* = 3.3, *P* = 0.0529), as was the relationship between marital status and telomere length (*F* = 2.0, *P* = 0.0967). Housing status was associated significantly with telomere length (*F* = 6.3, *P* = 0.0054). Individuals who reported that they were buying a house and those reporting that they were renting had shorter telomeres than participants in the other housing status category (ie. not buying or renting).

Physical activity, indexed according to MET-minutes, was associated positively with telomere length (*F* = 6.1, *P* < 0.0024). Participants who reported high levels of physical activity had significantly longer telomeres than those labeled sedentary, or those with low activity, or moderate activity, after controlling for the covariates. Similarly, body mass index (BMI) was a significant predictor of telomere length (*F* = 4.1, *P* = 0.0094). Specifically, normal weight individuals had significantly longer telomeres than obese participants, and also those missing BMI data, after controlling for the covariates. For alcohol use, telomere length did not differ among abstainers, moderate drinkers, and heavy drinkers (*F* = 1.2, *P* = 0.3129). However, telomere length differed significantly across categories of coffee intake (*F* = 4.5, *P* = 0.0104). Adults consuming high levels of coffee had significantly longer telomeres compared to those with moderate or low intakes. Those reporting no coffee intake and those with high intakes did not differ in telomere length. Cigarette smoking, indexed by pack years, was treated as a continuous variable, and with the other variables controlled, telomeres were 2.8 base pairs shorter for each pack year reported (*F* = 5.9, *P* = 0.0221).

## Discussion

The present investigation examined the relationship between caffeine consumption and telomere length in a large, nationally representative sample of U.S. adults, ages 20–84. A secondary objective was to study the association between coffee consumption and telomere length. Results showed that as caffeine intake increased, telomere length tended to decrease in U.S. adults, signifying accelerated aging. Conversely, as coffee intake increased, telomere length tended to increase, suggesting decelerated aging.

On the surface, it might be assumed that caffeine intake and coffee consumption are essentially the same variable. They are not. The present investigation illustrates this and several other studies also show this directly or indirectly. For example, in a large prospective study by Lopez-Garcia et al, data sets of the Health Professionals Follow-up Study and the Nurses’ Health Study were combined. Coffee intake was inversely associated with all-cause mortality, however, decaffeinated coffee consumption was also inversely related to the all-cause death rate [2], suggesting that factors other than caffeine were driving the relationship. Lopez-Garcia et al conclude, “Our data also suggest that this association was due to components in coffee other than caffeine” (p. 911). Later the authors [2] state that the healthful components of coffee “can counterbalance some of the potential harmful effects of caffeine, such as the acute stimulation of the release of epinephrine, a potent inhibitor of insulin activity, and the acute increase in blood pressure and homocysteine levels” (p. 912) [5–7].

In another large cohort study, Loftfield et al determined that coffee consumption was inversely associated with all-cause mortality, as well as death from heart disease, chronic lower respiratory disease, diabetes, and other disorders. However, the researchers concluded that “similar findings were observed for decaffeinated coffee and coffee additives” (p. 1010) [8]. In short, it appears that the favorable link between coffee and mortality is a function of factors in coffee, such as the phenolic compounds [30], increased insulin sensitivity [31], or reduced vulnerability to low-density lipoprotein oxidation [32], but not caffeine.

To date, it appears that only one other epidemiologic investigation has evaluated the relationship between caffeine intake and telomere length [33]. The primary objective of the Liu et al [33] investigation was to study the coffee and telomere length connection, but caffeine was also evaluated. Although telomere length tended to increase as coffee consumption increased, caffeine intake was not related to telomere length after adjusting for the covariates. Factors other than caffeine seem to be responsible for the longer telomeres found in coffee drinkers.

In the present investigation, the inverse relationship between caffeine intake and telomere length was dose-response and consistent. After adjusting for the covariates, adults with no caffeine intake had the longest telomeres and those with the highest caffeine intakes had the shortest. Moreover, the dose-response pattern prevailed across all of the coffee subgroups—coffee drinkers only, those reporting no coffee intake, and when all NHANES participants were studied together.

Comparing the relationships between classic public health concerns, such as obesity and smoking, to the link between caffeine intake and telomere length, provides meaningful reference points. Given the regression-based rate of telomere shortening was 15.3 base pairs per year of age, adults consuming 200 mg of caffeine per day would be projected to have 4.6 years of advanced cellular aging (70.8 ÷ 15.3 = 4.6). Similarly, using the same sample, obese adults had telomeres that were 82 base pairs shorter than normal weight individuals (Table 2), suggesting 5.4 years of accelerated biologic aging (82 ÷ 15.3 = 5.4). Likewise, adults reporting 25 pack-years of smoking had an estimated 4.6 years of increased cellular aging compared to non-smokers. Straightforward interpretation of the regression findings suggest that caffeine consumption accounts for a meaningfully higher rate of biologic aging in U.S. adults, particularly when the relationships between telomere length, obesity, and smoking are used as a gauge.

The inverse relationship between caffeine consumption and telomere length in U.S. adults could be a result of multiple factors. Because of the cross-sectional design of the present study, cause-and-effect conclusions are not warranted. First, a directionality problem is possible. Instead of A causing B, B could be causing A. Theoretically, shorter telomeres could lead to increased caffeine intake in U.S. adults. Because there is no evidence in the literature to support this pattern of reverse-causation, it is unlikely.

Another explanation for the observed inverse connection between caffeine intake and telomere length could be a “third variable,” resulting in a spurious relationship. To minimize this threat, statistical adjustments were made for a variety of sociodemographic and lifestyle covariates, and the results indicated that the relationship between caffeine consumption and telomere length was not explained by differences in these factors. Of course, it is possible that there are other “third variables,” not measured in the present study, which could account for the association between caffeine intake and telomere length in U.S. adults.

The present investigation had several limitations. First, the study design was cross-sectional. Hence, cause-and-effect conclusions are not warranted. Also, caffeine and coffee consumption were self-reported based on one 24-h recall. Therefore, measurement error could affect the outcome, although such error would likely weaken the associations between caffeine and coffee intake and telomere length, not strengthen them. Additionally, adults who consume caffeine may reflect a unique group who practice a different lifestyle than others. Further, premature aging is associated with a number of chronic conditions, including cardiovascular disease, chronic obstructive pulmonary disease, renal failure, Alzheimer’s disease, and others [34–36]. Many of these disorders are characterized by chronic inflammation [36]. Participants with and without these diseases were not identified nor controlled in the present study. Moreover, medication use often accompanies chronic disease. The effect of medications on telomere length is largely unknown. Therefore, the relationships uncovered in the present study could be partly explained by these or other conditions. Finally, Type I error is always a possibility. In short, the relationships between caffeine intake, coffee consumption, and telomere length could be a result of chance.

The present study also had multiple strengths. It appears to be one of the first investigations to evaluate the association between caffeine consumption and telomere length. Second, the sample was large (*n* = 5,826), multi-racial, and nationally representative of U.S. men and women, 20–84 years of age. The findings can be generalized to the U.S. adult population. Third, adjustments were made for differences in numerous potential confounding variables, including age, gender, race, marital status, education, housing arrangements, BMI, smoking, physical activity, coffee intake, and alcohol use. Lastly, measurement methods were validated and data collection was conducted by trained NHANES researchers.

## Conclusions

Caffeine intake is pervasive throughout much of the world. It has been linked to a number of beneficial and detrimental health consequences [1]. Unfortunately, much of the epidemiologic research on the effects of caffeine has focused on coffee intake, not caffeine. The present study, which investigated the relationships between caffeine and coffee intakes and telomere length, shows that as caffeine intake increases, telomeres tend to be shorter in U.S. adults. On the other hand, this investigation indicates that as coffee intake increases, telomeres tend to be longer. Because telomere length is a biomarker of the senescence of cells, the present findings suggest that cell aging may be accelerated in U.S. adults as caffeine intake increases, but may be decelerated as coffee consumption increases. Given the magnitude and importance of these relationships, additional research is warranted.

## Abbreviations

BMI: Body mass index
CDC: Centers for Disease Control and Prevention
CVD: Cardiovascular disease
DNA: Deoxyribonucleic acid
MET: Metabolic equivalent
mg: Milligram
NCHS: National Center for Health Statistics
NHANES: National Health and Nutrition Examination Survey
PSU: Primary sampling unit
SE: Standard error
U.S: United States
